# The Clinicopathological Features and Prognosis in Patients With Papillary Renal Cell Carcinoma: A Multicenter Retrospective Study in Chinese Population

**DOI:** 10.3389/fonc.2021.753690

**Published:** 2021-09-21

**Authors:** Baoan Hong, Huimin Hou, Lingxiao Chen, Zhi Li, Zhipeng Zhang, Qiang Zhao, Xin Du, Yuan Li, Xiongjun Ye, Wanhai Xu, Ming Liu, Ning Zhang

**Affiliations:** ^1^Key Laboratory of Carcinogenesis and Translational Research (Ministry of Education/Beijing), Department of Urology, Peking University Cancer Hospital & Institute, Beijing, China; ^2^Department of Urology, Beijing Hospital, National Center of Gerontology, Institute of Geriatric Medicine, Chinese Academy of Medical Sciences, Beijing, China; ^3^Department of Urology, Xiangya Hospital, Central South University, Changsha, China; ^4^Department of Urology, The Second Xiangya Hospital, Central South University, Changsha, China; ^5^Urology and Lithotripsy Center, Peking University People’s Hospital, Beijing, China; ^6^Department of Urology, The 4th Affiliated Hospital of Harbin Medical University, Harbin, China

**Keywords:** carcinoma, papillary renal cell carcinoma, clinical features, pathological features, prognosis

## Abstract

**Objective:**

The purpose of this study was to compare the clinicopathological characteristics of type 1 and type 2 papillary renal cell carcinoma (PRCC) and to explore the prognostic factors of PRCC in the Chinese population.

**Methods:**

A total of 242 patients with PRCC from five Chinese medical centers were retrospectively included. From them, 82 were type 1 PRCC and 160 were type 2 PRCC. Clinicopathological features and oncologic outcomes were reviewed. The Kaplan–Meier analysis and log-rank test were performed to describe the progression-free survival (PFS) and overall survival (OS). Univariate and multivariate Cox proportional hazards regression models were used to analyze the prognostic factors of PRCC.

**Results:**

Of the 242 patients, the average age at surgery was 55.3 ± 13.1 years. The mean tumor size was 5.1 ± 3.1 cm. Compared with type 1 PRCC patients, type 2 PRCC patients had a larger tumor size and were more likely to undergo radical nephrectomy. Besides, type 2 PRCC patients had higher tumor stage (*p* < 0.001) and WHO International Society of Urological Pathology (WHO/ISUP) grading (*p* < 0.001). Furthermore, tumor necrosis was more common in type 2 PRCC than type 1 PRCC (*p* = 0.030). The Kaplan–Meier survival analysis showed that the PFS and OS of type 1 PRCC patients were significantly better than those of type 2 PRCC patients (*p* = 0.0032 and *p* = 0.0385, respectively). Univariate analysis showed that tumor size, surgical procedures, pT stage, WHO/ISUP grading, and microvascular invasion were significant predictors of PFS and OS for type 2 PRCC patients. In the multivariate analysis, only pT stage (*p* = 0.004) and WHO/ISUP grading (*p* = 0.010) were the independent risk factors. Among type 2 PRCC patients with pT1 stage, no significant difference was found in PFS and OS between the partial nephrectomy and radical nephrectomy groups (*p* = 0.159 and *p* = 0.239, respectively).

**Conclusion:**

This multi-institutional study reveals the significant differences in clinicopathological variables and oncologic outcomes between type 1 and 2 PRCC. For type 2 PRCC in pT1 stage, the prognosis of partial nephrectomy is not inferior to that of radical nephrectomy, and nephron-sparing surgery can be considered.

## Background

Papillary renal cell carcinoma (PRCC) is one of the most common renal cell carcinoma (RCC), second only to clear cell RCC, accounting for 15% to 20% of all RCCs (1). It has significant heterogeneity, mainly for the different histopathological subtypes, biological behaviors, and clinical outcomes. The initial study by Delahunt and Eble reported that PRCC can be categorized into type 1 and type 2 based on histomorphological and immunohistochemical characteristics (2). Typically, type 1 tumor cells are cubic, are basophilic in cytoplasm, and have small nuclei, and the papillary structure is covered by a single layer of cells; while type 2 tumor cells are tall columnar, are rich in eosinophilic cytoplasm, and have obvious nucleoli, and the papillary structure is covered by pseudostratified cells (3).

According to previous studies, the two PRCC subtypes have differences in clinicopathological characteristics and prognosis. PRCC type 2 has a more advanced stage, a higher nuclear grade, and a worse prognosis than type 1 (4–6). However, on the contrary, some studies reported that PRCC subtyping had no effect on oncologic outcomes (7–10). Therefore, whether the histologic subtypes affect the prognosis are still controversial. In addition, most of these studies were carried out in Western populations. There is still a lack of studies to reveal the different clinical significance between PRCC subtypes in the Chinese population.

Hence, in the present study, we conducted a multicenter retrospective study that included a large sample of Chinese PRCC patients. The goal was to better understand the clinicopathological features and clinical outcomes of type 1 and type 2 PRCC in the Chinese population, further reveal prognostic factors, and provide clinical guidance.

## Methods

### Patient Population and Clinicopathological Features

Under the supervision of the Institutional Review Board, the data of RCC patients who underwent radical nephrectomy (RN) or partial nephrectomy (PN) at five medical centers in China from 2010 to 2020 were reviewed. Patients with incomplete clinicopathological data or lost to follow-up were eliminated. A total of 242 patients pathologically diagnosed with PRCC were included. Of these patients definite in classification, 82 patients were type 1 PRCC and 160 cases were type 2.

Clinical features included patient gender, age, initial symptoms, tumor location, and surgical procedures. Pathological parameters included PRCC subtypes, tumor size, pathologic T (pT) stage, grade, microvascular invasion, necrosis, and sarcomatoid differentiation. The samples were graded according to 2016 WHO International Society of Urological Pathology (WHO/ISUP) grading system. The pathological staging was determined according to the 2018 American Joint Committee on Cancer staging manual.

The main outcomes concerned were progression-free survival (PFS) and overall survival (OS). In this study, PFS was defined as the duration from surgery to local recurrence or distant metastasis, and OS was defined as the duration from surgery to death from any cause. Surviving patients were censored at the last follow-up.

### Statistical Analysis

The independent samples *t*-test was used to compare the continuous variables, and the χ^2^ test was used to compare the categorical variables. The impacts of the PRCC subclassification and clinicopathological features on PFS and OS were described with the Kaplan–Meier curve and compared with the log-rank test. Associations of clinical and pathological features with PFS and OS were analyzed using univariate and multivariate Cox proportional hazards regression models and presented with the HR and 95% CI. Ten variables were included in the stepwise selection analysis. Statistical analysis was performed with SPSS version 22.0 (IBM Corporation, Armonk, NY), and two-sided *p* < 0.05 was defined as statistically significant.

## Results

In this study, 177 (73.1%) of the patients were male and 65 (26.9%) of them were female. The male-to-female ratio was 2.7 to 1. The average age at surgery was 55.3 ± 13.1 years (ranging from 15 to 82). Of the 242 patients, 68.6% of patients were asymptomatic and were detected incidentally through physical examination. The clinical symptoms of the remaining cases included flank pain (14.5%), hematuria (12.0%), and other symptoms such as fever or renal dysfunction (5.0%). The mean tumor size was 5.1 ± 3.1 cm. The pathologic stage was T1a in 111 cases (45.9%), T1b in 62 (25.6%), T2 in 35 (14.5%), T3 in 22 (9.1%), and T4 in 12 (5.0%). The WHO/ISUP grading was G1 in 35 cases (14.5%), G2 in 98 (40.5%), G3 in 91 (37.6%), and G4 in 18 (7.4%).

The baseline clinicopathological characteristics of the PRCC patients are listed in **Table 1**. As shown in the table, RN was more common for type 2 PRCC patients than for type 1 patients (70.6% *vs.* 32.9%; *p* < 0.001). Compared with type 1 PRCC tumors, type 2 PRCC tumors were larger (*p* < 0.001). Besides, type 2 PRCC patients were likely to have a more advanced tumor stage (*p* < 0.001) and higher WHO/ISUP grade (*p* < 0.001). Moreover, the incidence of tumor necrosis in type 2 PRCC is higher than that in type 1 PRCC (*p* = 0.030). Other clinicopathological characteristics (including gender, age, initial symptoms, tumor location, microvascular invasion, and sarcomatoid differentiation) had no significant differences between the two subtypes.

**Table 1:** Comparison of clinicopathological characteristics between type 1 and type 2 PRCC.

Characteristic	Type 1 (n = 82)	Type 2 (n = 160)	*p*
**Gender**			0.535
Male	62	115	
Female	20	45	
**Age (years)**	55.3 ± 11.7	55.2 ± 13.8	0.988
**Initial symptoms**			0.154
No symptoms	63	103	
Hematuria	5	24	
Lumbago	10	25	
Other	4	8	
**Tumor location**			0.782
Left	41	77	
Right	41	83	
**Surgical procedures**			<0.001
Partial nephrectomy	55	47	
Radical nephrectomy	27	113	
**Tumor size (cm)**	4.1 ± 2.5	5.6 ± 3.3	<0.001
**Pathologic T stage**			<0.001
T1a	55	56	
T1b	13	49	
T2	10	25	
T3	4	18	
T4	0	12	
**WHO/ISUP grade**			<0.001
G1	25	10	
G2	36	62	
G3	17	74	
G4	4	14	
**Microvascular invasion**			0.179
No	77	141	
Yes	5	19	
**Tumor necrosis**			0.030
No	68	111	
Yes	14	49	
**Sarcomatoid differentiation**			0.665
No	81	156	
Yes	1	4	

WHO/ISUP, WHO International Society of Urological Pathology.

**Figure 1:**
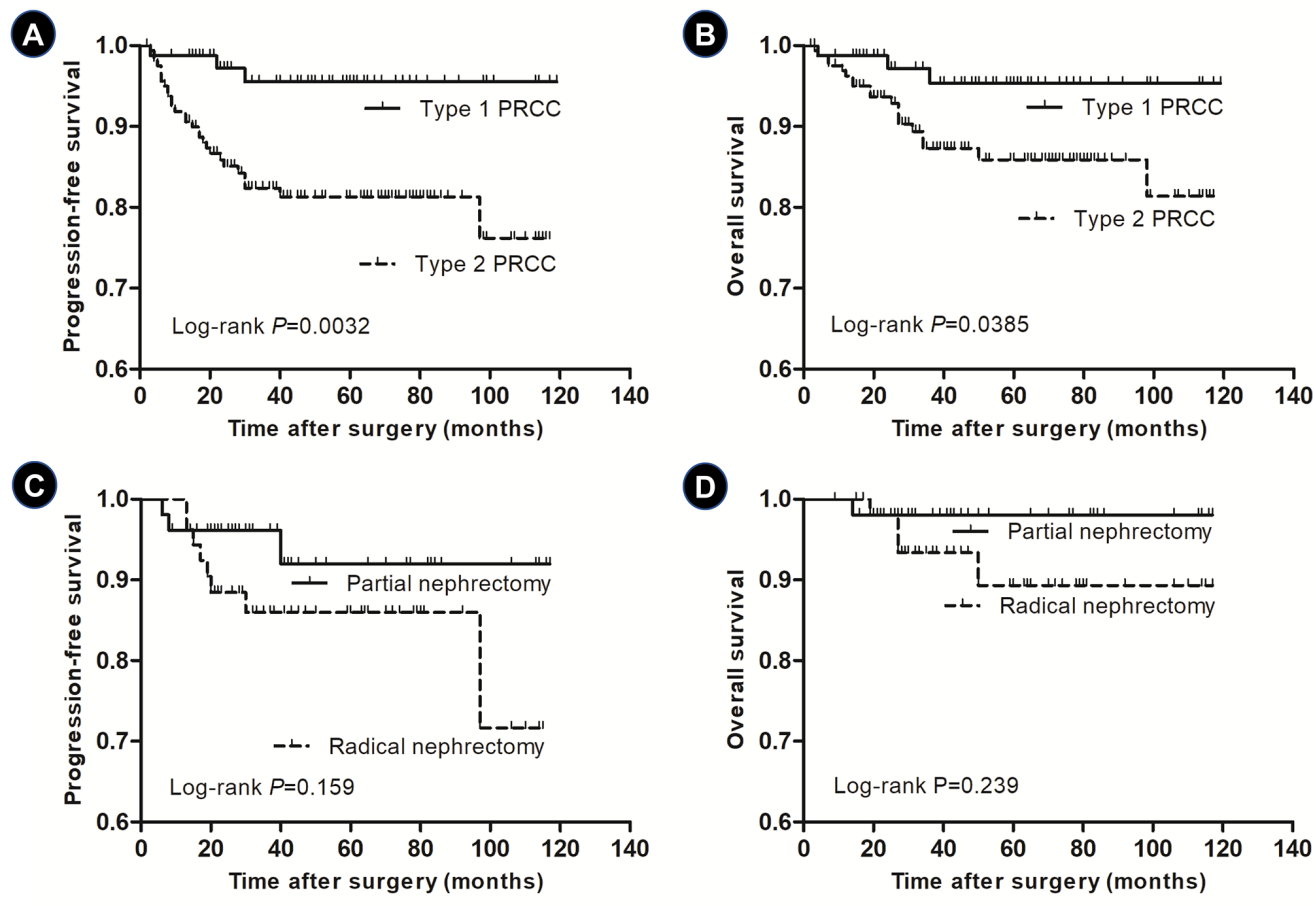
**(A)** Comparison of progression-free survival (PFS) between type 1 and type 2 papillary renal cell carcinoma (PRCC) (*p* = 0.0032). **(B)** Comparison of overall survival (OS) between type 1 and type 2 PRCC (*p* = 0.0385). **(C)** Comparison of PFS of type 2 PRCC patients with pT1 stage receiving partial nephrectomy and radical nephrectomy (*p* = 0.159). **(D)** Comparison of OS of type 2 PRCC patients with pT1 stage receiving partial nephrectomy and radical nephrectomy (*p* = 0.239).

The average follow-up duration was 48.9 months. To the end of follow-up, a total of 31 patients underwent disease progression, and 22 cases had died. The Kaplan–Meier plots for PFS indicated that type 2 PRCC patients had a lower probability for PFS than type 1 PRCC (*p* = 0.0032, **Figure 1A**). The estimated 1-year PFS for type 1 and type 2 PRCC patients reached 98.8% and 91.9%, respectively; and their 5-year PFS was 95.5% and 81.3%, respectively. In addition, the risk of death in patients with type 2 PRCC is significantly higher than that of patients with type 1 PRCC (*p* = 0.0385, **Figure 1B**). The 1-year OS of type 1 and type 2 PRCC reached 98.8% and 96.2%, respectively; and their 5-year OS was 95.3% and 85.9%. Thus, the Kaplan–Meier survival analysis demonstrated that the PFS and OS of type 1 PRCC were significantly better than those of type 2 PRCC patients.

Given the fact that statistical power was weakened by the limited progression or death events among type 1 PRCC, a subgroup univariate and multivariate Cox regression analysis of factors predictive of PFS and OS in type 2 PRCC patients was further performed. Univariate analysis showed that tumor size (*p* = 0.015), surgical procedures (*p* = 0.010), pT stage (*p* < 0.001), WHO/ISUP grading (*p* < 0.001), and microvascular invasion (*p* = 0.001) were significantly associated with PFS (**Table 2**). However, gender, age, tumor location, tumor necrosis, and sarcomatoid differentiation had no significant influence on PFS. According to multivariate analysis, only pT stage (*p* = 0.004) and WHO/ISUP grading (*p* = 0.010) were the independent risk factors for PFS of type 2 PRCC patients (**Table 2**). In univariate analysis, tumor size, surgical procedures, pT stage, WHO/ISUP grading, and microvascular invasion were significant predictors of OS (**Table 3**). Furthermore, stepwise multivariate analysis showed that pT stage (*p* = 0.007) and WHO/ISUP grading (*p* = 0.031) were independent predictors of OS (**Table 3**). Overall, of the various significant variables in univariate analysis, only pT stage and WHO/ISUP grading were independent risk factors for both PFS and OS in multivariate analysis.

**Table 2:** Univariate and multivariate Cox regression analyses of factors predictive of progression-free survival in type 2 PRCC patients.

Variable	Univariate analysis			Multivariate analysis		
	HR	95% CI	*p*	HR	95% CI	*p*
Gender (male *vs.* female)	1.111	0.472–2.616	0.809	–	–	–
Age (>55 *vs.* ≤55)	1.582	0.728–3.437	0.246	–	–	–
Tumor location (left *vs.* right)	1.505	0.712–3.182	0.285	–	–	–
Tumor size (>5.8 cm *vs.* ≤5.8 cm)	2.617	1.205–5.683	0.015	1.032	0.450–2.364	0.941
Surgical procedures (RN *vs.* PN)	4.877	1.472–16.160	0.010	2.562	0.713–9.210	0.149
T stage (III/IV *vs.* I/II)	6.692	3.177–14.097	<0.001	3.879	1.559–9.649	0.004
WHO/ISUP grade (G3/4 *vs.* G1/2)	8.546	2.570–28.416	<0.001	5.275	1.500–18.551	0.010
Microvascular invasion (yes *vs.* no)	4.138	1.820–9.408	0.001	1.356	0.496–3.709	0.553
Tumor necrosis (yes *vs.* no)	1.095	0.495–2.421	0.823	–	–	–
Sarcomatoid differentiation (yes *vs.* no)	2.186	0.296–16.171	0.444	–	–	–

PRCC, papillary renal cell carcinoma; RN, radical nephrectomy; PN, partial nephrectomy; WHO/ISUP, WHO International Society of Urological Pathology.

**Table 3:** Univariate and multivariate Cox regression analyses of factors predictive of overall survival in type 2 PRCC patients.

Variable	Univariate analysis			Multivariate analysis		
	HR	95% CI	*p*	HR	95% CI	*p*
Gender (male *vs.* female)	1.014	0.364–2.821	0.979	–	–	–
Age (>55 *vs.* ≤55)	1.077	0.430–2.695	0.875	–	–	–
Tumor location (left *vs.* right)	1.255	0.510–3.092	0.621	–	–	–
Tumor size (>5.8 cm *vs.* ≤5.8 cm)	5.224	1.732–15.753	0.003	1.706	0.534–5.451	0.368
Surgical procedures (RN *vs.* PN)	9.531	1.272–71.431	0.028	3.346	0.403–27.754	0.263
T stage (III/IV *vs.* I/II)	8.425	3.305–21.479	<0.001	4.489	1.506–13.382	0.007
WHO/ISUP grade (G3/4 *vs.* G1/2)	18.848	2.508–141.618	0.004	9.701	1.235–76.171	0.031
Microvascular invasion (yes *vs.* no)	4.225	1.602–11.142	0.004	1.671	0.542–5.148	0.371
Tumor necrosis (yes *vs.* no)	1.072	0.407–2.823	0.888	–	–	–
Sarcomatoid differentiation (yes *vs.* no)	4.410	0.575–33.809	0.153	–	–	–

PRCC, papillary renal cell carcinoma; RN, radical nephrectomy; PN, partial nephrectomy; WHO/ISUP, WHO International Society of Urological Pathology.

Of the 160 type 2 PRCC patients, 57 patients received PN and 103 cases received RN. The pathologic stage was T1 in 110 cases (68.8%), T2 in 22 (13.8%), T3 in 16 (10.0%), and T4 in 12 (7.5%). The average follow-up duration was 47.9 months. For type 2 PRCC patients with pT1 stage, stratified analysis showed that no significant difference in PFS (*p* = 0.159, **Figure 1C**) and OS (*p* = 0.239, **Figure 1D**) was found between PN and RN groups.

## Discussion

PRCC accounts for the largest subset of non-clear cell RCC, while its overall incidence is not high (11). At present, there is still a lack of multicenter large-scale study about PRCC, and further exploration of the clinicopathological features and prognosis of PRCC is helpful for understanding this type of RCC. In 1976, Mancilla-Jimenez et al. (12) retrospectively analyzed the clinicopathological data of 224 cases of RCC including 34 cases of PRCC and proposed it as an independent subtype of RCC. In 1997, Delahunt and Eble (2) classified PRCC into two morphologically different types according to cytological structural characteristics. As reported, type 1 PRCC is more common than type 2 PRCC in Western population with a 2~3:1 ratio (13–15). However, in this study, the distribution between type 1 and type 2 PRCC was approximately 1:2, which was contrary to that reported in Western population. On the other hand, Ha et al. (16) reported a multi-institutional study of 274 Korean patients with PRCC, of which 118 had type 1 PRCC and 156 had type 2 PRCC. Thus, the high incidence of type 2 PRCC may be a distinct characteristic of the Asian population.

As reported, PRCC accounted for approximately 10%–14% of RCC in Western population. However, the incidence of PRCC was reported to be approximately 5.2%–5.6% in Japanese and Korean populations and 1.9–7.5% in Chinese population (5, 16, 17). In this study, PRCC accounted for 4.3% of RCC, which was significantly lower than the incidence reported in the West, but a similar incidence was reported in Japan, Korea, and China. However, whether the low incidence of PRCC is a characteristic of Asian population deserves further investigation. In terms of clinical symptoms at first diagnosis, most of patients in this study were asymptomatic (68.6%). Patients who presented symptoms accounted for 31.4%, and type 2 PRCC patients were more common. Type 2 PRCC is highly aggressive and prone to progression, often with early symptoms such as hematuria and flank pain.

In this PRCC cohort, patients with pT1 account for 71.5%, and patients with G2/G3 account for 78.1%. Moreover, studies had reported that PRCC patients with pT1 account for 70%–80%, and those with G2 and G3 accounted for 36%–43% and 32%–51%, respectively (13, 16). Therefore, the pathological staging of PRCC is mostly early stage, mainly T1 stage, and majority of the pathological grades are grade 2 and grade 3. Earlier studies indicated that prognosis between the two subclassifications of PRCC was different. Pignot et al. (4) reported that type 2 PRCC was associated with a later stage and a higher grade, and the incidence of microvascular invasion was higher (*p* < 0.001). Delahunt and Eble summarized that type 2 PRCC had a higher stage and grade than type 1, which was associated with worse prognosis (2). In this study, we revealed that type 2 patients had a more advanced pT stage and higher WHO/ISUP grading (*p* < 0.001) than had type 1. These pathological features of type 2 PRCC suggest that its tumor biological behavior may be more aggressive.

At present, the issue about the prognostic factors of PRCC patients is still controversial. For example, Pignot et al. (4) reported that tumor type, stage, grade, microvascular invasion, an absence of foam cells, the presence of sarcomatoid cells, and tumor necrosis were prognostic factors of PRCC, of which tumor type and TNM stage were independent prognostic factors. However, Ha et al. (16) demonstrated that subclassification of PRCC was not a significant prognostic factor, while the pathologic T stage was an independent prognostic factor. Some studies reported that the biological behavior of type 2 PRCC is more aggressive than type 1, whereas others claimed that subclassification of PRCC did not affect the prognosis. In this study, the 1-year OS of type 1 and type 2 PRCC reached 98.8% and 96.2%, respectively, while the 5-year OS were 95.3% and 85.9%, indicating that the prognosis of type 2 was worse than that of type 1. Further univariate and multivariate analyses for type 2 PRCC indicated that tumor size, surgical procedures, pT stage, WHO/ISUP grading, and microvascular invasion were significant predictors of OS, while only the pT stage and WHO/ISUP grading were independent predictors of OS.

The surgical procedures play an important role in clinical treatment decisions. It was reported that nephron-sparing surgery is recommended as a priority for PRCC, and RN is preferable when nephron-saving surgery is not appropriate (18). Histologically, type 2 PRCC is more aggressive than type 1 PRCC, with higher pathological stage and grade, which may account for RN being recommended for type 2 PRCC patients. However, Bigot et al. (7) showed that histological subtyping had no impact on oncologic outcomes for PRCC patients receiving nephron-sparing surgery, which is suitable for localized PRCC. In the present study, stratified analysis showed that no significant difference in PFS (*p* = 0.159) and OS (*p* = 0.239) was found between PN and RN groups for type 2 PRCC patients with pT1 stage. As the study was retrospective, for tumors of the same stage, surgeons might choose different surgical methods according to the tumor location and the patient’s condition, which might lead to analytical bias. However, during the limited follow-up duration in this study, the prognosis of pT1 patients with type 2 PRCC who underwent PN was not worse than that of RN. The results may suggest that multifocality is not common in low-stage type 2 PRCC and PN could be considered for patients with stage T1 who are suspected to be type 2 PRCC preoperatively.

Although this is a multi-institutional study on Chinese PRCC patients, potential limitations need to be noticed. It is a retrospective study, and the follow-up duration was limited, so the strength of evidence needs to be further improved. Additionally, patients who did not have access to complete clinicopathological data were excluded, which might lead to selection bias. Moreover, the preferences and experience of surgeons and pathologists are difficult to adjust, which can also cause bias.

## Conclusions

Our findings indicated that the PFS and OS of type 1 PRCC were significantly better than those of type 2 PRCC. Compared with type 1 PRCC, type 2 PRCC had higher tumor stage and WHO/ISUP grading. Furthermore, tumor necrosis was more common in type 2 PRCC than type 1. In multivariate analysis of type 2 PRCC, pT stage and WHO/ISUP grading were independent predictors of PFS and OS. Interestingly, among type 2 PRCC with pT1 stage, no significant difference was observed in PFS and OS between PN and RN groups.

## Data Availability Statement

The raw data supporting the conclusions of this article will be made available by the authors, without undue reservation.

## Ethics Statement

The studies involving human participants were reviewed and approved by Institutional Review Board of Peking University Cancer Hospital & Institute. The patients/participants provided their written informed consent to participate in this study.

## Author Contributions

Conceptualization: NZ, ML, YL, WX, and XY. Data curation: BH, HH, LC, ZL, ZZ, QZ, and XD. Formal analysis: BH, HH, LC, ZL, ZZ, QZ, and XD. Funding acquisition: NZ, ML, and YL. Project administration and supervision: NZ, ML, YL, WX, and XY. Original draft writing: BH and NZ. All authors contributed to the article and approved the submitted version.

## Funding

This study was supported by Capital’s Funds for Health Improvement and Research (Grant No. 2020-2-1024); Capital Clinical Characteristic Application Research Project (Grant No. Z171100001017201); the Natural Science Foundation of Beijing (Grant No. 7212010); and Beijing Xisike Clinical Oncology Research Foundation (Grant No. Y-2019AZZD-0369).

## Conflict of Interest

The authors declare that the research was conducted in the absence of any commercial or financial relationships that could be construed as a potential conflict of interest.

## Publisher’s Note

All claims expressed in this article are solely those of the authors and do not necessarily represent those of their affiliated organizations, or those of the publisher, the editors and the reviewers. Any product that may be evaluated in this article, or claim that may be made by its manufacturer, is not guaranteed or endorsed by the publisher.
